# A Carbamoylase-Based Bioassay for the Detection of Paralytic Shellfish Poisoning Toxins

**DOI:** 10.3390/s20020507

**Published:** 2020-01-16

**Authors:** Mariana Raposo, Maria João Botelho, Sara T. Costa, Maria Teresa S. R. Gomes, Alisa Rudnitskaya

**Affiliations:** 1 CESAM and Chemistry Department, University of Aveiro, 3810-193 Aveiro, Portugal; micr@ua.pt (M.R.); mtgomes@ua.pt (M.T.S.R.G.); 2 IPMA, Portuguese Institute for the Sea and Atmosphere, 1449-006 Lisbon, Portugal; mjbotelho@ipma.pt (M.J.B.); sara.dacosta@ipma.pt (S.T.C.); 3 CIIMAR, Interdisciplinary Centre of Marine and Environmental Research, University of Porto, 4050-123 Porto, Portugal

**Keywords:** carbamoylase, paralytic shellfish toxins, potentiometric sensor, gonyautoxin 5, decarbamoylsaxitoxin, enzymatic assay

## Abstract

Out of control proliferation of toxic phytoplankton, called harmful algal blooms (HABs), have a significant economic impact on bivalve aquaculture and harvesting in coastal waters. Some phytotoxins, such as paralytic shellfish toxins (PSTs), are of concern due to the life-threatening symptoms they can cause. Development of rapid and low-cost screening tools would be a welcome addition to the laboratory methodologies employed in routine monitoring programs. However, most of the assays and biosensors for the screening of PSTs, are restricted to a single target, saxitoxin (STX), which is the most potent PST. The present study aimed at developing an assay for the detection of N-sulfocarbamoyl PST—GTX5, which is one of the most abundant toxins in bivalves during *G. catenatum* blooms as found on the Portuguese coast. Enzymatic assay employing PSTs’ transforming enzyme—carbamoylase—was proposed. Carbamoylase was extracted and purified from the surf clam *S. solida*. Carbamoylase displayed similar specificity to both carbamate (STX) and N-sulfocarbamate toxins (GTX5 and C1+2) converting them into decarbamoyl saxitoxin (dcSTX) and decarbamoyl gonyautoxins 2+3 (dcGTX2+3), respectively. The enzymatic assay involved hydrolysis of GTX5 by carbamoylase and quantification of the product of enzymatic reaction, dcSTX, using a potentiometric chemical sensor. A potentiometric sensor with plasticized PVC membrane that displayed sensitivity to dcSTX and selectivity in the presence of GTX5 was employed. Enzymatic assay allowed determination of GTX5 in the concentration range from 0.43 to 3.30 µmolL^−1^, which encompasses levels of GTX5 in contaminated bivalve extracts with toxicities above PSTs regulatory limits. The feasibility of the carbamoylase-based potentiometric assay for detection of GTX5 was demonstrated.

## 1. Introduction

Most coastal countries are affected by out of control proliferation of microalgae—Harmful algal blooms (HABs) [1,2]. Some of the species of these microalgae produce toxins, which can affect marine organisms and can be accumulated by bivalves. Subsequent consumption of contaminated seafood may provoke shellfish poisoning in humans [3]. Due to the unpredictability of the occurrence of HABs, routine surveillance programs of toxins in commercial bivalves and toxic phytoplankton species in seawater near bivalve harvesting areas were established in EU countries, including Portugal. Three groups of toxins, classified according the symptoms they cause in humans, are included in the monitoring program: diarrheic shellfish toxins (DSTs), paralytic shellfish toxins (PSTs) and amnesic shellfish toxins (ASTs), besides some other lipophilic toxins [4].

PSTs comprise about 30 3,4,6-trialkyl tetrahydropurine compounds with different combinations of hydroxyl, carbamoyl and carbamoylsulfate substituents [5]. All PSTs have the same mode of action, the inhibition of the voltage-gated sodium channel in excitable cells, though their toxicity varies significantly. Toxic episodes caused by the paralytic toxins are less frequent in Portuguese coastal waters compared to the other types of toxins [6], however, these compounds are of particular concern due to the severe neurological symptoms they impart to humans. In extreme cases, paralytic shellfish poisoning can be fatal, with overall mortality ranging between 2–14% [5,7].

Until 2005, the official reference method for the detection of PSTs in bivalves was the mouse bioassay (MBA), which involved injection of bivalve extracts into a mouse followed by observation of the animal’s condition over a predefined time frame. MBA is not only unethical and cruel, but it is also slow, expensive, and technically inadequate. Besides, it lacks selectivity, as it can respond to other toxic substances, such as metals, and display insufficient detection limits, which are close to regulatory values [8]. Ongoing efforts to discontinue the use of MBA in monitoring programs in EU have been confronted with several obstacles. One of them is the implementation of the alternative analytical method for PST detection, Liquid Chromatography with Fluorometric Detection (LC-FLD) [9], which requires investment in expensive apparatus and highly skilled personnel to operate it. Furthermore, LC-FLD involves laborious and time-consuming sample preparation. In this context, development of less expensive and complex assays and probes for PST detection and quantification is of practical interest for both routine and out of the laboratory shellfish safety screening.

Several biosensors and immunoassays based on monoclonal and polyclonal antibodies have been proposed for PSTs’ detection [10,11]. The most commonly used detection platforms include surface plasmon resonance (SPR) biosensors [12,13,14,15,16], enzyme labeled immunosorbent assay (ELISA) [13,17,18], flow cytometric assay [19] and optical [20,21] and electrochemical [22] biosensors.

Assays and sensors making use of the PSTs’ action in blocking voltage gated sodium channel, a protein present in several types of cells, have been proposed, including neuroblastoma cell-based assays [23,24] and impedimetric biosensors [25,26], and sodium channel or receptor based assays [27,28], and biosensors with detection by quartz microbalance [29] and sodium-selective electrode [30].

Nerve cell and receptor-based methods have the important advantage of producing toxicity estimates which are well correlated with MBA, making unnecessary use of the toxicity equivalency factors. However, these methods involve laborious preparation procedures, use radioactive isotopes in the case of receptor based assays, display long response times and lack stability resulting in low reliability and reproducibility of measurements [23,27,31,32].

Antibody-based assays and biosensors display very high specificity and low detection but only to some of the known PSTs. Antibodies employed in these tools are mostly produced against saxitoxin and, thus, assays and biosensors possess low affinity to other PSTs, especially to toxins differing from saxitoxin in the epitope region such as the sulfocarbamoyl ones [33,34]. A combination of different antibodies in assays to achieve broader sensitivity has been suggested in order to overcome this limitation [18]. No antibodies to N-sufocarbamoyl toxins have been reported in the literature. However, PST outbreaks caused by dinoflagellate *Gymnodinium catenatum* generate toxin profiles in bivalves that primarily include N-sulfocarbamoyl and decarbamoyl toxins, while saxitoxin is typically absent [35], making existing antibody based assays inapplicable. While *G. catenatum* is less common compared to other PST-producing dinoflagellates, it has a worldwide distribution including the Gulf of California, Gulf of Mexico, Venezuela, Argentina, Korea, China, Japan, the Philippines, Tasmania, New Zealand, the Mediterranean and the Atlantic coasts of Spain, Portugal and Morocco [36,37].

Simultaneous quantification of four PSTs, STX, dcSTX, GTX5 and C1+2 using an electronic tongue comprising six potentiometric sensors was reported earlier [38]. However, two issues were identified in the previous work: higher errors of determination of sulfocarbamoyl toxins GTX5 and C1&2 due to the low selectivity of the sensors to these toxins in the presence of dcSTX and STX, and the necessity to use cleaned up bivalve extracts for the measurements. Extract cleanup is a laborious and time-consuming procedure that would be desirable to avoid. The rational to develop an enzymatic assay for detection of GTX5 was, firstly, improvement of the quantification precision of this toxin, and, secondly, elimination of the bivalve extract cleanup step by using a sensor capable to detect dcSTX (the enzymatic reaction product) in bivalve extracts without cleanup [38].

The purpose of this study was thus the development of an enzymatic assay with potentiometric detection for determination of PSTs typically related to *G. catenatum* blooms. The proposed assay relies on the use of an enzyme capable of hydrolyzing the carbamate and sulfocarbamoyl groups of PSTs. PST hydrolysis attributable to the enzymatic activity was observed in several clam species [39,40,41] and two enzymes with different specificity, named sulfocarbamoylase and carbamoylase, were extracted from two East-Asian clam species, *Peronidia venulosa* and *Mactra chinensis*, respectively [42,43]. Similar toxin-transforming action has been observed in the crude extracts of bivalve species from the Portuguese coast including *Spisula solida* [44,45], which was used as the source of enzyme in the present study. As far as we know, no enzyme-based biosensors for PST determination were previously reported in the literature.

## 2. Materials and Methods

### 2.1. Reagents

Sodium hydrogen phosphate and dihydrogen phosphate, hydrochloric acid and sulfuric acids, sodium hydroxide, aniline, multiwalled carbon nanotubes (MWCNT), tetrahydrofuran (Chromasolv), ammonium formate, acetic acid, TRIS (tris(hydroxymethyl) aminomethane (BioPerformance Certified) were from Sigma Aldrich Química, S.L. (Algés, Portugal), ammonium sulfate and aprotinin were from VWR International—Material de Laboratório, Lda (Alfragide, Portugal); bestatin was from Santa Cruz B6iotechnology Inc. (Heidelberg, Germany). Sodium dodecyl sulfate (SDS), methanol and acetonitrile (HPLC grade) was from Riedel-de-Haën (Seelze, Germany). Sodium hydroxide, sodium chloride and hydrogen peroxide were purchased from Merck Millipore (Algés, Portugal). Pierce™ BCA Protein Assay Kit was from Thermo Fisher Scientific (Porto Salvo, Portugal). All reagents were p.a. (for analysis) grade unless stated otherwise.

Solutions of PSTs, namely saxitoxin (STX), decarbamoyl saxitoxin (dcSTX), gonyautoxin-5 (GTX5), N-sulfocarbamoyl toxins C1+2 and decarbamoyl gonyautoxin 2+3 (dcGTX2+3), were certified reference material from the Institute for Marine Biosciences, National Research Council (Halifax, NS, Canada). High molecular weight polyvinyl chloride, dibutyl phthalate, potassium tetrakis(4-chlorophenyl)borate and octadecyl 4-formylbenzoate were Selectophore^®^ grade from Sigma Aldrich. Screen-printed electrodes (SPE) with gold working and auxiliary electrodes and silver reference electrode were from DropSens (Asturias, Spain). Ultrapure water (18 MΩcm^−1^) was used for all solution preparation.

### 2.2. Extraction and Purification of PST Transforming Enzymes

Enzyme was extracted from digestive gland and crystalline style tissues dissected from surf clam (*S. solida*) specimens purchased at a local market. Extraction and purification procedures were adopted from [42,43]. Briefly, digestive glands were homogenized with equal volume of 100 mmolL^−1^ phosphate buffer (pH 7) at 4 °C. After centrifugation at 4 °C and 1500× *g* for 15 min, followed by 3900× *g* for 15 min, and then 8000× *g* for 60 min, and filtration of the supernatant through 10- followed by 5-µm membrane filters, crude extract was obtained. Crude extract was submitted to salting out procedure by adding solid ammonium sulfate to a saturation of 20%, stirring for 2 h and centrifuging at 10,000× *g* for 30 min at 4 °C. The 60% ammonium sulfate fraction was collected and dissolved in 50 mL of 50 mmolL^−1^ phosphate buffer (pH 7) containing 1.5 molL^−1^ of ammonium sulfate, and filtered through 5- and 0.65-µm membrane filters. Crystalline style was homogenized at 4 °C with approximately 10 volumes of 50 mmolL^−1^ phosphate buffer containing 0.5 molL^−1^ of ammonium sulfate, 5 mg L^−1^ of aprotinin, 5 mg L^−1^ of bestatin, and 0.1 mmolL^−1^ of EDTA. After centrifugation at 4 °C and 550× *g* followed by 7500× *g* for 30 min and filtration of the supernatant through 10-µm membrane, crude extract was obtained.

The 60% ammonium sulfate fraction of digestive gland extract and crude crystalline style extract were independently loaded onto an octyl Sepharose 4 fast flow column as described below. Prior to purification of digestive gland extract, the column was equilibrated with 50 mmolL^−1^ phosphate buffer (pH 7) containing 1.5 molL^−1^ of ammonium sulfate. The column was eluted stepwise with five column volumes of 50 mmolL^−1^ phosphate buffer containing 0.7 molL^−1^, than 0 molL^−1^ ammonium sulfate and ending with deionized water at a flow rate of 4 mL min^−1^. Prior to purification of crystalline style extract, the column was equilibrated with 50 mmolL^−1^ phosphate buffer (pH 7) containing 0.5 molL^−1^ of ammonium sulfate and was eluted stepwise with five column volumes of 50 mmolL^−1^ phosphate buffer and deionized water at a flow rate of 2.5 mL min^−1^. The 50 mmolL^−1^ phosphate buffer eluates obtained from both purification procedures were concentrated by ultrafiltration using a 30 K filter and stored at −80 °C prior to use.

### 2.3. Enzyme Characterization

Enzymatic activities of crude extracts and purified enzyme towards STX, GTX5 and C1+2 were evaluated using the following procedure: 1 µmolL^−1^ of toxin certified material solution was mixed with 100 µL of enzyme extract in 50 mmolL^−1^ phosphate buffer and incubated during 3 h at 25 °C.

Specific activity of carbamoylase, present in the extracts, towards those three toxins was determined using discontinuous assays. A series of solutions have been prepared by mixing 50 µL of purified enzyme solution, 50 mmolL^−1^ phosphate buffer and 1 µmolL^−1^ of toxin solution. Reaction mixtures were incubated for 10, 20, 30, 45 and 60 min at 25 °C. One munit (mU) enzyme was defined as the amount of enzyme that catalyzes the reaction of 1 nmol of substrate per minute.

The influence of experimental conditions on the enzymatic reaction was evaluated by varying temperature and composition of buffer solutions. Enzymatic reaction was run at 14, 20, 25 and 30 °C. The following buffers were tested: phosphate buffer (pH 7) with concentrations of 0.25, 1, 13, 25 and 50 mmolL^−1^, Tris buffer (pH 7) with concentrations of 0.25, 1, 2 and 50 mmolL^−1^ and borate buffer (pH 9) with concentration of 50 mmolL^−1^. Reaction mixtures were prepared by mixing 100 µL with 1 µL of toxin solutions and incubated during 3 h at 25 °C. Effect of the temperature was tested using enzyme extracts in 50 mmolL^−1^ phosphate buffer.

Enzymatic reactions were stopped by addition of 10 µL of 10 molL^−1^ acetic acid, after which they were frozen at −18 °C till further analysis of PSTs by LC-FLD. Assays were run in triplicate.

Enzyme concentration in extracts was determined using a protein quantification kit, based on the Smith method, and used according to the instructions of the manufacturer.

The molecular weight of carbamoylase was determined by 12% SDS-PAGE as described by Laemmli [46].

### 2.4. PSTs’ Quantification

Quantification of PSTs in the enzymatic reaction mixtures was done using LC-FLD according to the official AOAC method [47]. The LC analysis was carried out using a Hewlett-Packard/Agilent model 1050 quaternary pump, Model 1100 in-line degasser, autosampler, column oven, and model 1200 fluorescence detector (Agilent Tehcnologies, Santa Clara, CA, USA). The Hewlett-Packard Chemstation software performed data acquisition and peak integration. The procedure used in the oxidation of PSTs was based on [13] with a procedural modification for determination of N-sulfocarbamoyl and decarbamoyl compounds. The toxin oxidation procedure, chromatographic conditions and details in PSTs quantification are described elsewhere [48,49].

### 2.5. Sensor Fabrication and Potentiometric Measurements

Potentiometric sensors were constructed using commercially available SPE according to the procedure described in [50]. Briefly, surface of SPE working electrode was rinsed with ethanol and water, after which it was cleaned electrochemically by sweeping potential between −0.2 and +1.2 V at 50 mV/s in 50 mmolL^−1^ sulfuric acid three times. A solid contact layer was prepared by electropolymerizing aniline in the presence of carbon nanotubes, using deaerated aqueous aniline solution 50 mmolL^−1^, hydrochloric acid 1 molL^−1^, SDS 0.1 molL^−1^ and 0.17 g L^−1^ MWCNTs by cycling potential for 40 cycles between −0.23 and +0.85 V at 50 mV/s. Sensor was washed with ultrapure water, conditioned in 10 mmolL^−1^ hydrochloric acid for 2 h and dried. An EZstat-Pro EIS instrument (NuVant Systems Inc., Crown Point, IN, USA) was used for electrochemical experiments. A Ag/AgCl (KCl 3 molL^−1^) reference electrode was used.

Membrane mixture had the following composition: PVC (33% *w*/*w*), dibutyl phthalate (66% *w*/*w*), octadecyl 4-formylbenzoate (1% *w*/*w*) and potassium tetrakis(4-chlorophenyl)borate (0.5% *w*/*w*). Membrane components were dissolved in tetrahydrofuran and 10 µL of membrane mixture were deposited on top of the solid contact and spun at 700 rpm for 5 min using DELTA 10TT spin coating (Süss-MicroTec, Garching, Germany). This procedure was repeated twice, after which the sensor was left to dry at room temperature for 24 h. Prior to use, the sensor was conditioned in ultrapure water for 2 h.

Calibration measurements were made in dcSTX standard solutions prepared by diluting the certified dcSTX solutions in 1 mmolL^−1^ phosphate buffer (pH 7). Concentrations of dcSTX ranged from 0.1 to 6.8 µmolL^−1^. A drop of the solution was applied on top of the sensor electrodes and potential was recorded 5 min later, in order to ensure stable readings. Custom-made high input impedance digital voltmeter (Sensor Systems LLC., St. Petersburg, Russia) was used for potentiometric measurements. Voltmeter was connected to a PC for data acquisition. Sensor potentials were measured vs. SPE own pseudo-reference electrode. Between measurements sensor was washed with ultrapure water until stable potential readings were reached.

Enzymatic assays were carried out by mixing 20 µl of enzyme solution (3,9 mg mL^−1^) and GTX5 solutions with toxin concentrations varying from 0.52 to 6.7 µmolL^−1^. Mixed solutions were incubated at 25 °C for 17 h and measured using the potentiometric sensor.

## 3. Results and Discussion

### 3.1. Carbamoylase Characterization

**Figure 1:**
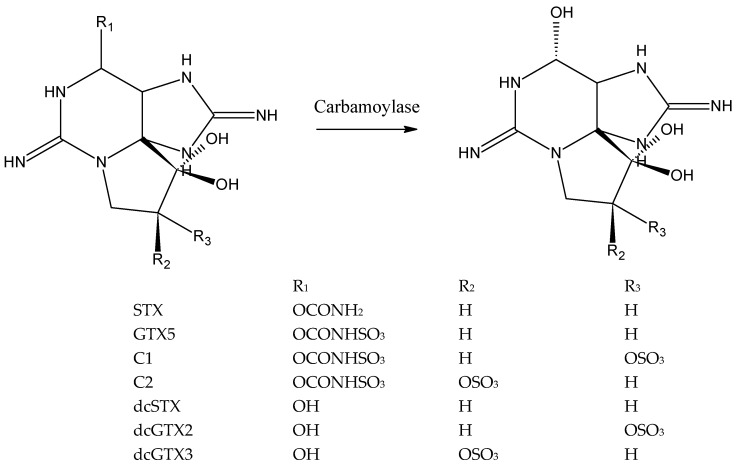
Reaction of carbamoylase with PSTs: Cleavage of carbamoyl or sulfocarbamoyl moiety (R1). The following transformation take place: STX → dcSTX, GTX5 → dcSTX, C1+2 → dcGTX2+3.

The purpose of this study was to develop an assay for the detection of N-sulfocarbamoyl PSTs based on the enzyme carbamoylase. Carbamoylase catalyzes hydrolysis of the carbamate (R_1_ = –CONH_2_) or N-sulfocarbamoyl moiety (R_1_ = –CONHSO_3_) of PSTs, turning them into the corresponding decarbamoyl analogues, dcSTX in the case of STX or GTX5, dcGTX2 in the case of C1 and dcGTX3 in the case of C2 (Figure 1) [43,46].

Carbamoylases can use as substrates any carbamate and N-sulfocarbamoyl toxins, though with varying specificity, which is also dependent on the origin of the enzyme. Carbamoylase is produced by several clam species. It has been extracted and purified from *M. chinensis* [42], and its action was documented for *P. staminea* [39,40] and *S. solida* [41,44,45]. *S. solida* was selected as the source of carbamoylase in the present study.

It is important to note that the toxins C1 and C2, and dcGTX3 and dcGTX3 are stereoisomers with respect to the sulfate group at C11 (R_2_ and R_3_, Figure 1). Both pairs of stereoisomers, C1 and C2, and dcGTX2 and dcGTX3, are present as mixtures in bivalves and in the commercially available reference materials are racemic mixtures: C1+2 contains 77% of C1 and 23% of C2, and dcGTX2+3 contains 77% of dcGTX2 and 23% of dcGTX3 [51]. Furthermore, isomers are detected together by the reference method LC-FLD, as a single product is formed after oxidation. Thus, analytical detection by the sensor was also envisaged without stereoisomer distinction, and therefore, each pair of isomers was considered as one compound in this work, i.e., C1+2 and dcGTX2+3.

Digestive gland and crystalline style were identified as the bivalve tissues with highest activity of carbamoylase in earlier reports [42,43] and, thus, were selected for enzyme extraction. Table 1 shows the enzymatic activity towards STX of crude and purified extracts of digestive gland and crystalline style tissues. While all extracts contained enzyme capable of transforming STX, the highest activity was observed in the purified extract of crystalline style. This result is in agreement with the anatomical distribution of toxin transforming enzymes that was reported for other clam species [42,43]. Further experiments were performed using purified enzyme extract from crystalline style.

The specific activity of the enzyme purified from crystalline style towards STX, GTX5 and C1+2 determined using a discontinuous assay and the substrate consumption percentages after 1 h of incubation are presented in the Table 2. Carbamoylase transformed all three toxins with affinities not statistically different from each other, which is consistent with literature data, though direct comparison is difficult as substrate consumption percentages over longer periods of time are reported. Carbamoylase obtained from Atlantic surf clam was reported to convert both carbamate and N-sulfocarbamoyl toxins with a faster conversion rate of N-sulfocarbamoyl ones. Conversion of 95% of STX and 99% of GTX5 after 12 h of incubation (no tests were run with C1+2 by the authors) was previously reported [45]. In another work [41], conversion of 100% of C1+2 and GTX5 after 1 h of incubation was observed, while 100% of STX was converted only after 6 h. Carbamoylase extracted from the Japanese clam *M. chinensis* converted 83% and 92% of STX and GTX5, respectively, and only 13% of C1+2 after 24 h of incubation [42].

**Figure 2:**
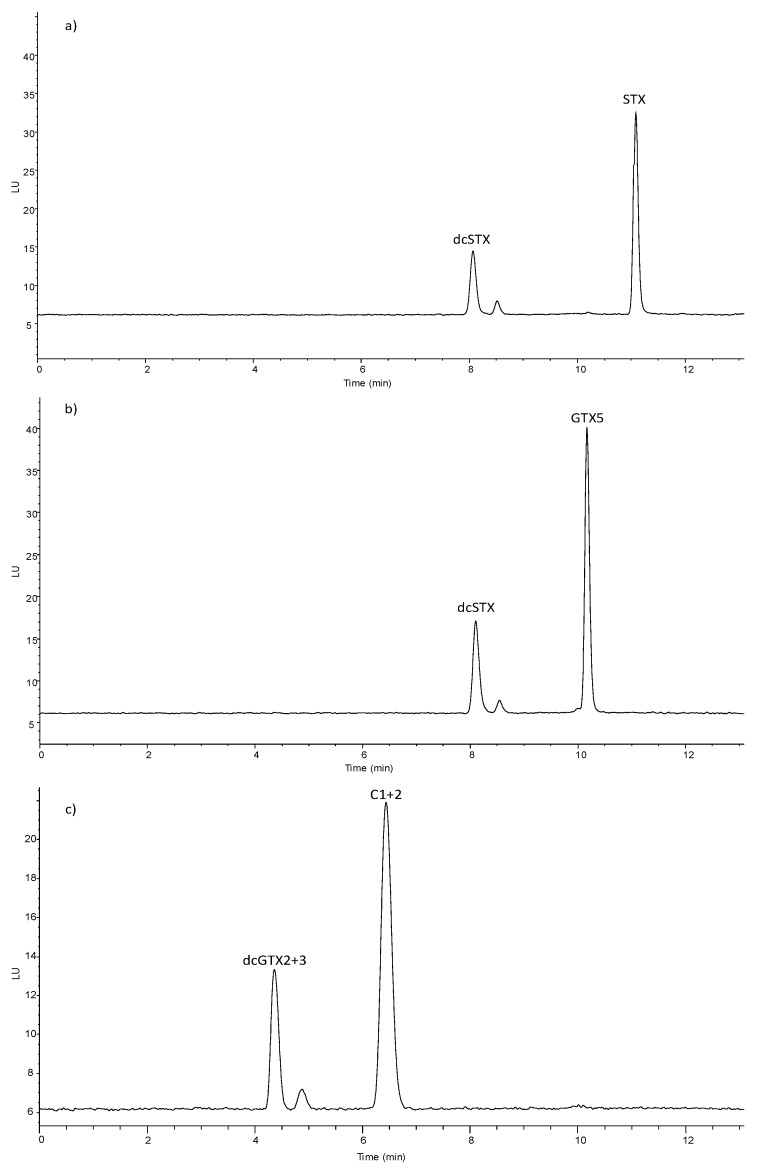
LC-FLD chromatograms obtained analyzing the reaction mixture of carbamoylase with toxin solutions STX (**a**), GTX5 (**b**) and C1+2 (**c**).

The mechanism of the enzymatic reaction, i.e., cleavage of the carbamate or sulfocarbamoyl moiety as depicted in Figure 1, is consistent with LC-FLD results of analysis of enzyme incubated with STX, GTX5 and C1+2 (Figure 2). In the enzymatic assay a decrease of the concentration of the substrate, STYX, GTX5 or C1+2 was observed together with the appearance of the enzymatic reaction products, dcSTX and dcGTX2+3.

**Figure 3:**
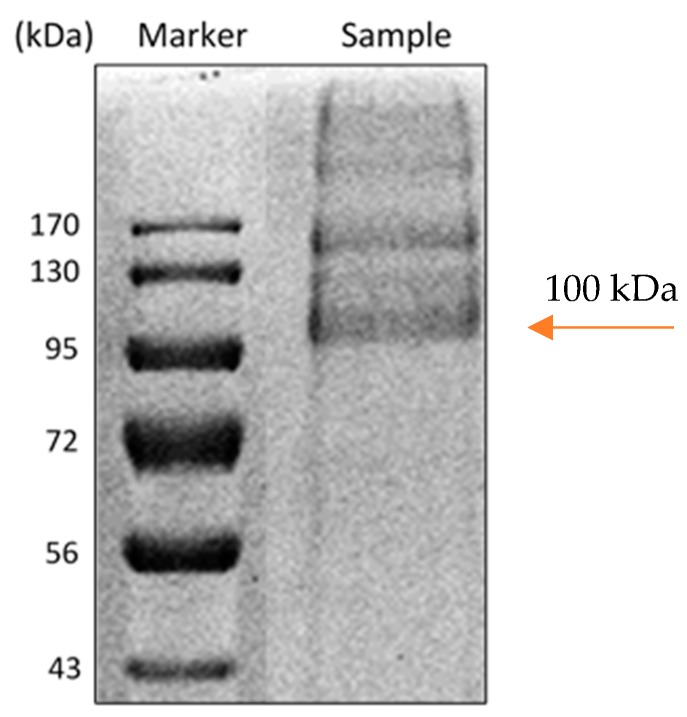
SDS-PAGE image of carbamoylase: The lane Marker is molecular weight standards, the lane Sample is purified carbamoylase. Arrow indicates the 100 kDa molecular weight of the enzyme.

Molecular weight of the enzyme was estimated to be ca. 100 kDa by SDS-PAGE electrophoresis (Figure 3), which is close to the value of 94 kDa reported for *M. chinensis* carbamoylase [42].

Due to its broad specificity, carbamoylase can be used in an assay for the detection of all three studied PSTs. Given that STX is typically absent in bivalves from Portuguese coast, carbamoylase can be used for detection of two N-sulfocarbamoyl toxins, GTX5 and C1+2. As decarbamoylated toxins resulting from hydrolysis of GTX5 and C1+2 are different, using a potentiometric sensor selective to one of them can impart selectivity to the assay. We opted for developing an assay targeting GTX5 with potentiometric detection of its hydrolysis product, dcSTX, as will be discussed below. GTX5 is one of the main PSTs detected on the Portuguese coast, which may account for up to 50 mol.% of total PSTs in bivalves [35].

### 3.2. Effect of Experimental Conditions on Enzymatic Activity

**Figure 4:**
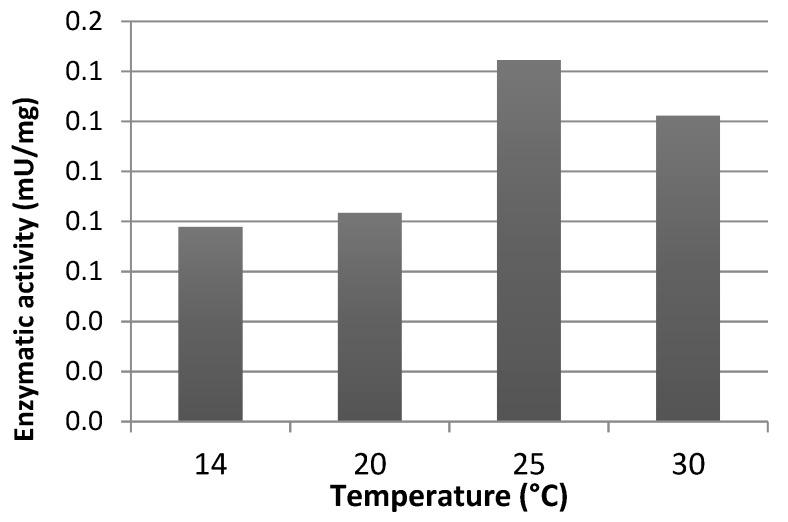
Effect of the temperature on carbamoylase activity. GTX5 was used as a substrate.

Effect of experimental conditions such as temperature, and a buffer composition, concentration and pH on carbamoylase activity was investigated using GTX5 as substrate. Maximum rate of enzymatic reaction was observed at 25 °C as shown in Figure 4.

Data about the influence of buffer concentration on enzymatic activity is indispensable for both the optimization of the enzymatic assay conditions and sensor selection. Therefore, pH levels that are relevant for the selection of the sensor as detector in the assay and composition and concentration of the buffer that are relevant for the functioning of the sensor were assessed. Detection of the enzymatic reaction products can target either decarbamoylated toxin, dcSTX, or ammonia ions formed after cleavage of the sulfocarbamoyl moiety of GTX5 [52]. One of the options of ammonia detection is amperometric, which requires pH of ca. 9 [53]. According to the literature, optimal pH for carbamoylase and sulfocarbamoylase is 7 [42,43]. Thus, it was tested if enzyme activity at pH 9 is much lower at pH 9 compared to optimal pH 7. For detection at pH 7, potentiometric detection using one of sensors sensitive to PSTs that was reported earlier can be used [50]. Though this sensor has high selectivity towards PSTs in the presence of sodium with selectivity coefficient logK_A/B_ = −2.9, large difference in the concentrations between toxin and sodium in the buffer mean that this selectivity may not be sufficient and sodium ions may affect sensor response. Thus, with the aim to avoid interference of the sodium on the sensor response, possibility to use a buffer with the pH 7 but without sodium, such as i.e., Tris, or decrease as much as possible concentration of phosphate buffer to decrease Na concentration, was evaluated.

**Figure 5:**
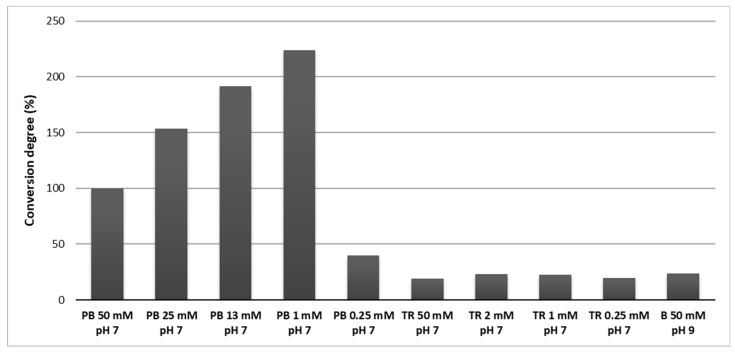
Substrate (GTX5) conversion degree by carbamoylase in buffers with different composition and pH. PB—phosphate buffer, TR—Tris buffer, B—borate buffer. Conversion degree values were normalized w.r.t. 50 mmolL^−1^ phosphate buffer.

Comparison of the enzymatic activity in different buffers is shown in the Figure 5. Use of Tris buffer resulted in lower enzymatic activity compared to phosphate buffer with the same pH and concentration (50 mmolL^−1^ and pH 7). Likewise, low enzymatic activity was observed in borate buffer with pH 9. While no difference in activity was observed in Tris buffer with different concentrations, the decrease of phosphate buffer concentration to 1 mmolL^−1^ brought an increase of the enzyme activity. However, below 1 mmolL^−1^ the enzyme activity sharply decreased. Thus, maximum activity of carbamoylase was observed in 1 mmolL^−1^ phosphate buffer with pH 7 at 25 °C.

### 3.3. Enzymatic Assay with Potentiometric Detection

A potentiometric sensor suitable as detector for carbamoylase assay for GTX5 determination should have high selectivity to the reaction product, dcSTX, in the presence of substrate, GTX5, or vice versa, and high selectivity in the presence of the buffer components such as sodium. Based on previous studies, potentiometric sensor with PVC/DBF/KTPB/octadecyl 4-formylbenzoate membrane was selected [50]. This sensor displayed a super-Nernstian response to STX and dcSTX with the slope of 69 ± 3 mV/pX, and the detection limit of 0.65 and 0.9 µmolL^−1^, respectively, in 1 mmolL^−1^ Tris buffer solution. This sensor also displayed high selectivity towards STX in the presence of Na ^+^ and K ^+^ with logK_sel_ −2.9 and −3.1, respectively, which should allow toxin detection in 1 mmolL^−1^ phosphate buffer. The sensor possessed slightly higher selectivity to dcSTX in the presence of STX, with logK_sel_ of 0.16. However, given that STX is typically absent in the PST profile produced by *G. catentum* blooms, it would not interfere with dcSTX detection.

**Figure 6:**
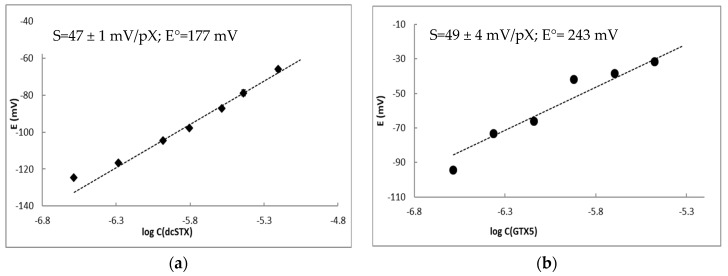
Sensor responses in the solutions of dcSTX (**a**) and in the solutions of dcSTX formed during enzymatic hydrolysis of GTX5 (**b**) prepared in 1 mmolL^−1^ phosphate buffer. Parameters of the sensor response in dcSTX solutions and enzymatic assay were calculated in the concentration ranges from 0.52 to 6.24 µmolL^−1^ (**a**) and from 0.43 to 3.34 µmolL^−1^ (**b**), respectively, are shown in the insets: S—slope of the electrode function with standard deviation and E°—standard potential.

Firstly, the response of the selected sensor to dcSTX, C1+2 and GTX5 under the optimal experimental conditions for enzymatic assay, i.e., using 1 mmolL^−1^ phosphate buffer as background, was evaluated. Sensor response to dcSTX was lower in 1 mmolL^−1^ phosphate buffer compared to the Tris buffer with the same concentration presenting a slope of the electrode function that decreased from 69 to 47 ± 1 mV/pX (Figure 6a). The sensor displayed no response to either GTX5 or C1+2 in phosphate buffer (data not shown), and therefore can be used for quantification of dcSTX in the presence of both GTX5 and sodium, meaning it can be used as a detector in enzymatic assays for GTX5 determination. If dcSTX is present in the sample, it can be determined using the same sensor prior to enzymatic assay.

The enzymatic assay was run in solutions with GTX5 concentrations ranging from 0.43 to 3.34 µmolL^−1^, which were measured using the potentiometric sensor after incubation. Response of the sensor to GTX5 concentration in the assay was found to be 49 mV/pX (Figure 6b). The slope obtained in the assay was not statistically different (*p* = 0.05) from the one obtained for dcSTX alone, confirming the possibility to determine GTX5 through the quantification of the product of its enzymatic hydrolysis. Standard potential of the sensor differed being higher in the case of the assay. This deviation can be related to the presence of the enzyme or other compounds in the enzyme solution added to the assay.

In order to be applicable to PSTs determination, the detection limit of the developed sensor needs to be below legal regulatory limits. The regulatory limit for PSTs is 800 μg of STX equivalents per kg of bivalve meat, where STX equivalents are calculated using toxicity equivalence factors for each toxin. Concentration of GTX5 corresponding to the regulatory limit is 2.7 µmolL^−1^ in bivalve extracts prepared according to the official method [50]. GTX5 accounts for approximately 20–40% of PSTs molar fraction detected in bivalves during toxicity episodes, which correspond to the toxin levels of 0.54–1.35 µmolL^−1^ in bivalve extract. As the developed assay allows quantification of GTX5 in the concentration range between 0.43 and 3.34 µmolL^−1^, it can be employed as a screening tool for this toxin in bivalve extracts. The main limitations of this enzymatic assay for practical applications are laborious procedure of enzyme extraction and purification, and relatively long assay time. In the future work assay time can be decreased by assessing intermediate incubation times and increasing enzyme concentration. However, no currently available method is very fast. For example, commercially available ELISA assay requires 3.5 h of incubation in total and involves several manipulations.

## 4. Conclusions

An enzymatic assay for the detection of one of the dominant PSTs in bivalves during *G. catenatum* blooms on the Portuguese coast, GTX5, was developed using PST transforming enzyme carbamoylase and a potentiometric chemical sensor. Carbamoylase was extracted from the crystalline style of the surf clam *S. solida* and found to hydrolyze carbamate (STX) and N-sulfocarbamoyl toxins (GTX5, C1+2) with similar specificity. Mechanism of the enzymatic reaction consisting in the cleavage of the carbamoyl or sulfocarbamoyl moiety of the toxin producing decarbamoylated PSTs, dcSTX in the case of STX and GTX5, and dcGTX2+3 in the case of C1+2. Optimization of the buffer concentration and composition with the aim to maximize enzymatic activity and avoid interference of the buffer constituents with the sensor response was carried out. Maximum enzymatic activity of carbamoylase was observed at 25 °C in 1 mmolL^−1^ phosphate buffer. Selectivity of the enzymatic assay to GTX5 was ensured by using potentiometric sensor selective to the product of GTX5 enzymatic hydrolysis, dcSTX. Enzymatic assay allows determination of GTX5 toxin in the concentration range from 0.43 to 3.34 µmolL^−1^, which corresponds to the levels of GTX5 observed in the extracts of bivalves with PST toxicity close to the regulatory limit.

## Figures and Tables

**Table 1:** Enzymatic activity towards STX of carbamoylase present in crude and purified extracts of surf clam digestive gland and crystalline style after 3 h of incubation.

Extract	Activity, mU/mg
Digestive gland, crude	1.1 ± 2.0
Digestive gland, purified	162.7 ± 1.1
Crystalline style, crude	33.5 ± 0.3
Crystalline style, purified	478.5 ± 0.5

**Table 2:** Specific enzymatic activity towards STX, C1+2 and GTX5 of carbamoylase purified from crystalline style of *S. solida* and substrate consumption (after 1 h of incubation).

Toxin	Specific Activity, mU/mg	Substrate Consumption, %
STX	38 ± 3	78
GTX5	37 ± 1	60
C1+2	35 ± 2	81
